# Windowed Joint Detection and Decoding with IR-HARQ for Asynchronous SCMA Systems

**DOI:** 10.3390/e25060930

**Published:** 2023-06-13

**Authors:** Mengsheng Guan, Min Zhu, Baoming Bai

**Affiliations:** 1 The State Key Laboratory of Integrated Services Networks, Xidian University, Xi’an 710071, China; 18011210001@stu.xidian.edu.cn (M.G.); bmbai@mail.xidian.edu.cn (B.B.); 2 Science and Technology on Communication Networks Laboratory, Shijiazhuang 050081, China

**Keywords:** IR-HARQ, RC-LDPC, SCMA, windowed joint detection and decoding

## Abstract

To improve the decoding performance of asynchronous sparse code multiple access (SCMA) systems over additive white Gaussian noise (AWGN) channels, this paper proposes a novel windowed joint detection and decoding algorithm for a rate-compatible (RC), LDPC code-based, incremental redundancy (IR) hybrid automatic repeat quest (HARQ) scheme. Since incremental decoding can exchange information iteratively with the detections made at previous consecutive time units, we propose a windowed joint detection and decoding algorithm. The extrinsic information exchanging process is performed between the decoders and the previous *w* detectors at different consecutive time units. Simulation results show that the sliding-window IR-HARQ scheme for the SCMA system outperforms the original IR-HARQ scheme with a joint detection and decoding algorithm. The throughput of the SCMA system with the proposed IR-HARQ scheme is also improved.

## 1. Introduction

In the field of next-generation communication systems for crowded scenarios, Non-Orthogonal Multiple Access (NOMA) technologies have emerged as promising solutions that meet access requirements with high reliability, low latency, and low costs. There are several available non-orthogonal access technologies, including power-domain non-orthogonal multiple access (PD-NOMA) [1], interleave division multiple access (IDMA) [2,3], multi-user shared access (MUSA) [4], pattern division multiple access (PDMA) [5], and sparse code multiple access (SCMA) [6]. These technologies allow multiple users to send messages simultaneously in the same frequency and code domains while maintaining orthogonality in other fields such as power allocation, interleavers, and patterns. SCMA is an advanced version of a low-density signature (LDS) that uses sparse code to modulate data symbols. SCMA systems use multi-dimensional constellation (MDS) mapping with a sparse indicator matrix, unlike LDS spreading and quadrature amplitude modulation (QAM). The receiver uses a decoder that employs a message-passing algorithm (MPA)-based multi-user detection (MUD). SCMA systems have better error-correction performance than LDS systems, with an overloading factor of up to 150% [6].

### 1.1. Related Work and Motivation of SCMA

Research on SCMA systems has primarily focused on codebook design [7,8,9,10], low-complexity detection [11,12,13], and iterative processing between detection and decoding. Mahmoud et al. proposed a unified framework for multiuser codebook designs in [7], which was later expanded upon in [8,9,10] with various design methods that offer better performance and lower complexity. For example, Zhang et al. introduced a codebook based on a uniquely decomposable constellation group (UDCG) in [8], while Munich et al. investigated the design of unequal error protection (UEP) codebooks in [9] and Bao et al. focused on codebook design for SCMA systems over Rayleigh fading channels in [10]. To simplify the decoder complexity, threshold-based MPA was introduced in [11], while [12] proposed a low-complexity decoding algorithm based on list sphere decoding. More recently, a novel algorithm based on alternating maximization with the exact penalty was proposed in [14] for the minimum Euclidean distance (MED) maximization problem. Zheng et al. proposed a simplified SCMA codebook with a separable structure in [15], which simplifies MED maximization when implementing a low-complexity detector. Based on the mother codebook, Lei et al. proposed a novel codebook design scheme to achieve better error performance and further explored an improved unified optimization to maximize the MED of each user in [16].

### 1.2. Related Work to SCMA Systems with HARQ Schemes

The SCMA system has seen an increase in usage and research, but few studies have examined its performance with the hybrid automatic repeat quest (HARQ) scheme. Shiomitsu et al. demonstrated the efficiency of this scheme in improving decoder performance for fifth-generation mobile communication technology (5G) transmission strategies in [17]. Building on this idea, the authors in [18] explore an SCMA-oriented HARQ scheme that enhances the reliability of new information by retransmitting correctly decoded codewords. However, this scheme may not be suitable for random access scenarios, as it requires immediate retransmission by users experiencing transmission failure in the current slot. To address this issue, ref. [19] presents an improved HARQ scheme dedicated to SCMA under grant-free random access scenarios. However, these two schemes have drawbacks, such as wasting resources and limited use of fixed-rate codes, which restricts the use of Chase combining (CC) HARQ for the SCMA system. Since the IR-HARQ scheme is a promising alternative to the CC-HARQ scheme for SCMA systems to improve throughput, Zhu et al. showed that the combination of rate-compatible (RC) codes and an IR-HARQ scheme performs well in [20,21]. Moreover, the binary RC-LDPC codes have been accepted as standard codes for the enhanced mobile broadband (eMBB) data channel of 5G communication in [22]. Thus, it is worth studying the performance of the RC-LDPC code-based IR-HARQ scheme for SCMA systems.

### 1.3. Main Contribution

This paper focuses on exploring the performance of an RC-LDPC code-based IR-HARQ scheme for SCMA systems. In [23], Zhu et al. explain the fundamental principle of this scheme and present a joint detection and decoding algorithm. However, the incremental decoder utilized in their work is solely related to the detection in the current transmission round, and not across different rounds. Consequently, there is room for optimization of the decoding algorithm’s efficiency and accuracy. To address this, we propose the transmission mechanism of the asynchronous IR-HARQ scheme, in which information can be sent during different transmission rounds based on each user’s channel conditions. This approach enables the incremental decoders and MUD to consider not only the extrinsic information at the current time unit but also at previous time units. Moreover, the windowed joint detection and decoding algorithm to simulate this scenario is introduced. The simulation indicates that the proposed scheme can enhance the bit error ratio (BER) performance and achieve better throughput over a wide range of SNRs.

The novelty and contributions of this paper can be summarized as follows:

- We build an asynchronous uplink SCMA system with the RC-LDPC code-based IR-HARQ scheme and present the transmission mechanism of the asynchronous IR-HARQ scheme.
- We propose a novel windowed joint detection and decoding algorithm with IR-HARQ for asynchronous SCMA systems.

### 1.4. Paper Organization

The rest of the paper is structured as follows. Section 2 briefly reviews the RC-LDPC codes, based on Kite codes, and introduces the proposed SCMA system model with the IR-HARQ scheme. Section 3 introduces the asynchronous IR-HARQ scheme, and then the RC-LDPC code-based IR-HARQ schemes for SCMA systems are detailed. The simulation results are presented in Section 4, and the conclusion is summarized in Section 5.

## 2. System Model

### 2.1. RC-LDPC Codes Based on Kite Codes

Kite codes, refs. [24,25] designed for noisy channels, are represented by C∈[∞,k;p]. These codes are defined by their *k* dimension and a p-sequence $p=\left(p_{1},p_{2},\ldots ,p_{t},\ldots \right)$, where $1<p_{t}<1$ for t≥0. A codeword c=(u,v) ∈ C[∞,k;p] consists of an information vector $u=\left(u_{1},u_{2},\ldots ,u_{k-1}\right)$ and a parity sequence $v=\left(v_{1},v_{2},\ldots ,v_{t},\ldots \right)$.

The prefix Kite code with length *n* can be represented as C[n,k] and is a systematic linear code with $r_{b}=n\times k$ parity bits. Its parity-check matrix H can be expressed as

(1) H=(Hu,Hv),

where Hu, corresponding to information bits, is a binary matrix of size r×k and Hv, corresponding to parity bits, is a dual-diagonal matrix of size r×r. It can be seen that Hu is a random-like binary matrix whose entries are governed by the p-sequence p:

(2) $$\;\mathrm{Hv}={\left[\begin{array}{ccccc} 1 & \; & & \; & \\ 1 & 1 & \; & & \\ \; & 1 & \ddots & \; & \\ \; & \; & \ddots & 1 & \\ \; & \; & & 1 & 1 \end{array}\right]}_{r\times r}.$$

Therefore, a Kite code is a unique LDPC code with a degree distribution determined by the p-sequence. We can use the sum-product algorithm during decoding. The construction of Kite codes allows for the generation of specific parity-check bits corresponding to each coding rate, with the higher-rate codes being able to serve as prefix codes of lower-rate codes. From Kite codes, RC-LDPC codes can be obtained, but in order to ensure that all prefix codes are good enough, the optimization of $p_{i}$, which determines the degree distribution of Kite code, is necessary.

Zhang et al. partitioned coding rates from 0.05 to 1 into 19 intervals in their work [25], assuming $p_{t}$ to be a step function of the coding rates. They enforced $p_{t}=q_{l}$ whenever *t* satisfied the coding rate k/(t+k)∈(0.05l,0.05(l+1)], utilizing 19 possible different values $q_{l},1\leq l\leq 19$. Therefore, the optimization process can be simplified to selecting $q=\left(q_{1},q_{2},\ldots ,q_{19}\right)$ for use in the greedy optimization algorithm. As $q_{l}$ represents a check-node degree distribution varying with coding rates, the RC-LDPC codes based on Kite codes (RC Kite codes) perform well after optimization. Zhang et al. also provided an optimization algorithm in [25] that achieved the approximate right-regular distribution.

### 2.2. Asynchronous IR-HARQ SCMA

We can consider an uplink SCMA system with *L* physical resources and each physical resource denoted by $U_{i}$, where $i\in \left\{1,2,\ldots ,L\right\}$. After mapping, it becomes a codeword $x_{i}$ of the SCMA codebooks. If we let $h_{i}$ represent the channel vector from the base station to the *i*-th physical resource, the received signal y can be expressed as

(3) $$y=\sqrt{\frac{E_{s}}{L}}\sum_{i=1}^{L}\mathrm{diag}\left(h_{i}\right)x_{i}+n.$$

**Figure 1:**
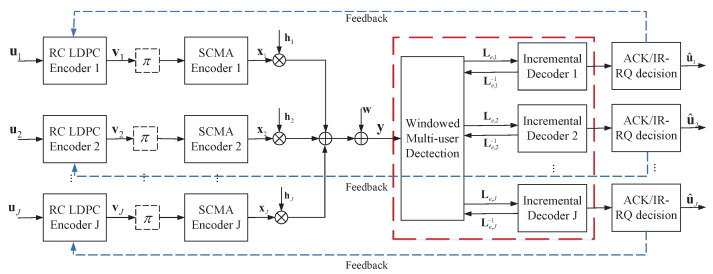
Block diagram of an asynchronous uplink SCMA system with the RC-LDPC code-based IR-HARQ scheme.

The diagram in Figure 1 illustrates the IR-HARQ strategy employed by the asynchronous uplink SCMA system with *J* users and *L* physical resources. Each user *j*, $j\in \left\{1,2,\ldots ,J\right\}$, encodes a data sequence $u_{j}$ of length *k* using an RC-LDPC code independently. The result is a codeword $v_{j}=\left(v_{j,1},v_{j,2},\ldots ,v_{j,N}\right)$ with each $v_{j,n}$ being either 0 or 1. To simplify, we denote the vector $\left(v_{j,1},v_{j,2},\ldots ,v_{j,N}\right)$ as ${\left\{v_{j,t}\right\}}_{t=1}^{N}$. The windowed joint detection decoder, boxed in the red line, is utilized at the receiver. Moreover, extrinsic information exchange between *J* decoders and the windowed detector is shown.

This paper discusses an SCMA system that involves 6 users sharing 4 resource elements. Each user has a codebook of size *M*, denoted by $X_{j}=\left\{a_{j}^{\left(i\right)}\subset C^{L},1\leq i\leq M\right\}$. The codebook consists of sparse signal vectors $a_{j}^{\left(i\right)}={\left\{a_{j}^{\left(i\right)}\left[\ell \right]\right\}}_{l=1}^{L}$, where *n* non-zero entities are positioned based on a sparse indicator vector $f_{j}$. The SCMA encoder is defined as $v_{j}={\left\{v_{j,t}\right\}}_{t=1}^{N}\to x_{j}={\left\{x_{j,t}\right\}}_{t=1}^{N}$ with $x_{j,t}\subset X_{j}$. The SCMA system uses an L×J indicator matrix $F=[f_{1},f_{2},\ldots ,f_{J}]$ to assign the vector $f_{j}$ to user *j*. Each $f_{j}$ is a binary vector of length *L* with row and column weight of $d_{f}$ and *n*, respectively. Therefore, each user transmits a coded symbol using n=2 complex symbols. The indicator matrix F is given as

(4) $$\;F_{4\times 6}=\left[\begin{array}{cccccc} 0 & 1 & 1 & 0 & 1 & 0\\ 1 & 0 & 1 & 0 & 0 & 1\\ 0 & 1 & 0 & 1 & 0 & 1\\ 1 & 0 & 0 & 1 & 1 & 0 \end{array}\right].$$

To learn more about the SCMA encoder, please refer to [7,26].

Assuming that all users’ signals are perfectly synchronized, we can combine Equations (3) and (4). The received superposed signals at the receiver during the symbol duration *t* can be written as

(5) $$y_{t}=\sum_{j=1}^{J}\mathrm{diag}\left(h_{j,t}\right)x_{j,t}+w_{t},$$

where $h_{j,t}$ is the channel gain vector and $w_{t}$ is the additive complex Gaussian noise vector. In addition, the correlation between each $h_{j,t}$ is determined by the coherence time of the channel, or Doppler spread. In situations with high mobility, the channel is rapidly time-varying and experiences multiple channel realizations in each retransmission round.

This paper considers that each transmitted block has a length of $N_{0}$ and that $D_{\max}$ represents the maximum number of transmissions, including the initial transmission and retransmissions. For a user *j*, $j\in \left\{1,2,\ldots ,J\right\}$, the retransmission request will be sent back to the sender if decoding fails. Upon receiving this request, the sender will send *I* redundancy bits specific to user *j* through the channel. This process will continue until decoding is successful or the maximum number of transmissions is reached.

## 3. Windowed Joint Detection and Decoding Algorithm

### 3.1. IR-HARQ Transmission Scheme

**Figure 2:**
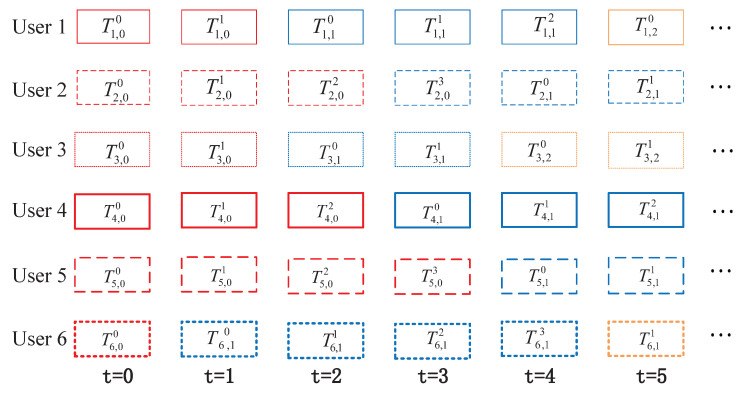
Transmission mechanism of asynchronous SCMA-oriented IR-HARQ scheme.

The detailed transmission mechanism of the asynchronous IR-HARQ scheme is shown in Figure 2. To begin, the first set of information symbols for each user is encoded using RC-LDPC codes, resulting in an initial block codeword sequence. If the receiver fails to decode this initial block, parity-check blocks related to it are sent over the channel and added to the initial block codeword to create a longer one. Without loss of generality, we assume that the block length for each transmission is the same under the IR-HARQ transmission scheme in 5G. The maximum number of transmissions for the initial block, including information symbols, is limited to four (i.e., $D_{\max}=4$). For user *j*, $j\in \left\{1,2,\ldots ,J\right\}$, the block $T_{j,i}^{d}$ represents the *d*th transmission ( $d\leq D_{\max}$) of length $N_{0}$ related to its initial block *i*, including the information symbols.

At the start, each user sends their initial block with a length of $N_{0}$ and a code rate of $R_{0}=k/N_{0}$. The SCMA encoder processes six blocks simultaneously and sends the resulting sequence through the channel. At the receiver, a joint detection and decoding algorithm is used. If all users successfully decode their blocks, they receive a new initial block for the next time unit. If decoding fails for some users, they receive a block of redundant parity bits corresponding to the previous initial block. Users who successfully decode the block receive a new initial block at the next time unit. This process is repeated for subsequent time units. In the provided diagram, users 1–4 decode their initial blocks with fewer than four transmissions, allowing them to move on to the second initial block. However, user 5 fails to decode their block in four transmissions, so a new initial block is transmitted on the fifth round. Similarly, user 6 fails to decode the second initial block in four transmissions, leading to the transmission of the third initial block in the sixth round.

### 3.2. Windowed Joint Detection and Decoding Algorithm

As described in Section 3.1, the incremental decoders at a specific time unit *t* depend not only on the detector at that time unit but also on the detectors at previous time units. This is because redundant parity bits are transmitted at different time units. To ensure accuracy, it is necessary to have iterations between the decoder and detector at the same time unit, as well as joint iterations between the decoder at the current time unit and the detectors at the previous time units.

**Figure 3:**
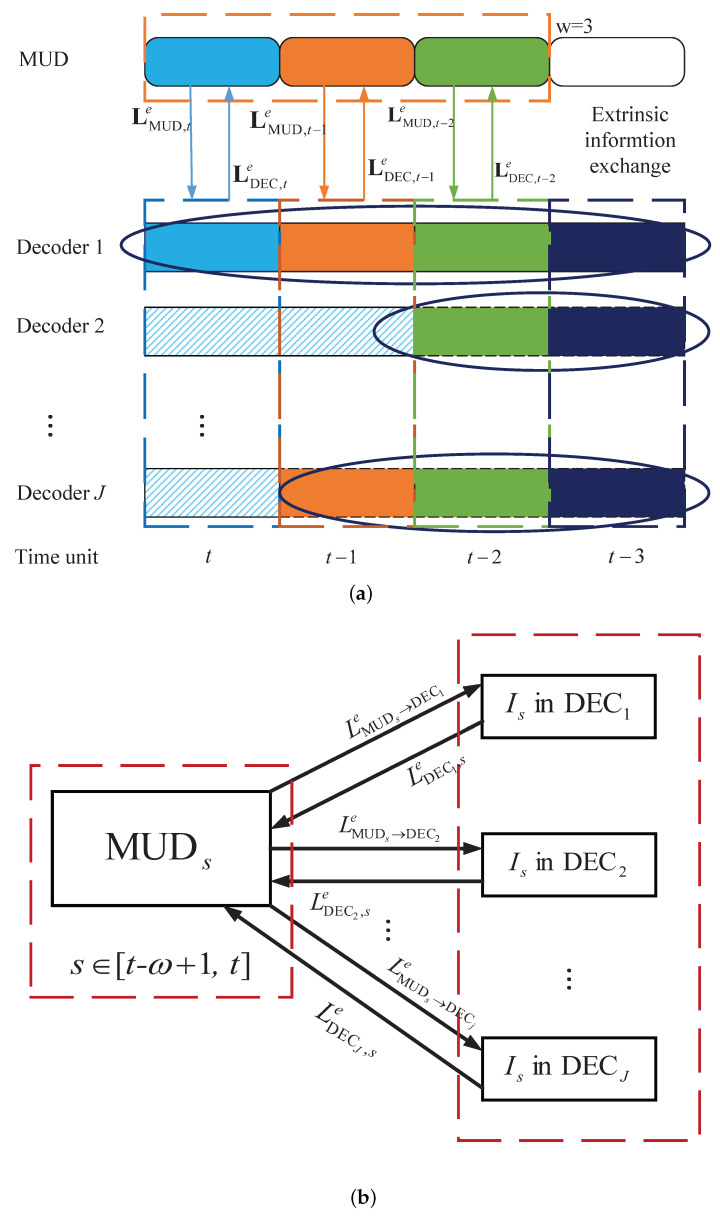
Decoding diagram with the windowed joint detection and decoding algorithm. (**a**) Windowed joint detection and decoding algorithm at different time units with *w* = 3. (**b**) The extrinsic information exchanged between ${\mathrm{MUD}}_{s}$, s∈[t−w+1,t] and ${\mathrm{DEC}}_{j}$, j=1,…,J at time unit *s*.

The decoding diagram of the windowed joint detection and decoding algorithm is shown in Figure 3. Assuming that the maximum number of transmissions for an information block is $D_{\max}=4$ and the window size is w=3. For simplicity, each decoder is represented by ${\mathrm{DEC}}_{j}$, j=1,…,J, and the block length received at a one-time unit is *I*. The slashed rectangles in the diagram depict the blocks that have been successfully decoded. The log-likelihood ratio (LLR) values of these decoded blocks remain constant during the iteration between the detector and decoder. The other colored rectangles represent the undecoded blocks. For example, and as shown in Figure 3, for ${\mathrm{DEC}}_{1}$, four blocks from time unit *t* to t−3 are decoded by an RC-LDPC decoder ${\mathrm{DEC}}_{1}$. The resulting extrinsic information $L_{{\mathrm{DEC}}_{1},t}^{e},L_{{\mathrm{DEC}}_{1},t-1}^{e},\ldots ,L_{{\mathrm{DEC}}_{1},t-w+1}^{e}$ is transmitted to detectors ${\mathrm{MUD}}_{t}$, ${\mathrm{MUD}}_{t-1}$,…, ${\mathrm{MUD}}_{t-w+1}$, respectively. Similarly, for ${\mathrm{DEC}}_{2}$, two blocks $\left({\mathrm{block}}_{t-2},{\mathrm{blcok}}_{t-3}\right)$ are decoded by an RC-LDPC decoder ${\mathrm{DEC}}_{2}$. The extrinsic information $L_{{\mathrm{DEC}}_{2},t-2}^{e}$ is transmitted to the ${\mathrm{MUD}}_{t-2}$. (Because the current window size is 3, $L_{{\mathrm{DEC}}_{2},t-3}^{e}$ will not be transmitted to ${\mathrm{MUD}}_{t-3}$.) The sliding window joint detection and decoding algorithm works as follows.

When the channel information is received at time unit *t*, the MUD (referred to as ${\mathrm{MUD}}_{t}$) performs the message-passing algorithm (MPA) detection and generates the extrinsic LLR information $L_{\mathrm{MUD},t}^{e}$. This information is then exchanged with the DECs.

After receiving the LLRs $L_{\mathrm{MUD},t}^{e}$ from ${\mathrm{MUD}}_{t}$, the decoders ${\mathrm{DEC}}_{t},t=1,2,\ldots ,J$, perform the incremental decoding (step 2 in Figure 3a). (The length of different decoders in the DEC is different because some users decode more new information symbols due to their good channel conditions.) The extrinsic LLRs , $L_{\mathrm{DEC}}^{e,w}=\left\{L_{\mathrm{DEC},t}^{e},L_{\mathrm{DEC},t-1}^{e},\ldots ,L_{\mathrm{DEC},t-w+1}^{e}\right\}$, are generated and delivered to ${\mathrm{MUD}}_{i}$, i=t,t−1,…,t−w+1 as the a priori information written by

(6) $$L_{\mathrm{MUD},s}^{a}=L_{\mathrm{DEC},s}^{e},\;\;\;s=t,t-1,\ldots ,t-w+1.$$

It is obvious that the window size is $w\leq D_{\max}$. Then, *w* MUDs work separately and deliver the resulting extrinsic information $L_{\mathrm{MUD}}^{e,w}=\left\{L_{\mathrm{MUD},t}^{e},L_{\mathrm{MUD},t-1}^{e},\ldots ,L_{\mathrm{MUD},t-w+1}^{e}\right\}$ to the DEC as

(7) $$L_{\mathrm{DEC},s}^{a}=L_{\mathrm{MUD},s}^{e},\;\;\;s=t,t-1,\ldots ,t-w+1.$$

Upon receiving the extrinsic LLRs from ${\mathrm{MUD}}_{i}$, i=t,t−1,…,t−w+1, the DEC performs incremental decoding and sends back the new extrinsic information to the *w* MUDs according to (6). This iteration process continues until the maximum number ${\mathrm{Iter}}_{\mathrm{out}}$ is achieved.

**Figure 4:**
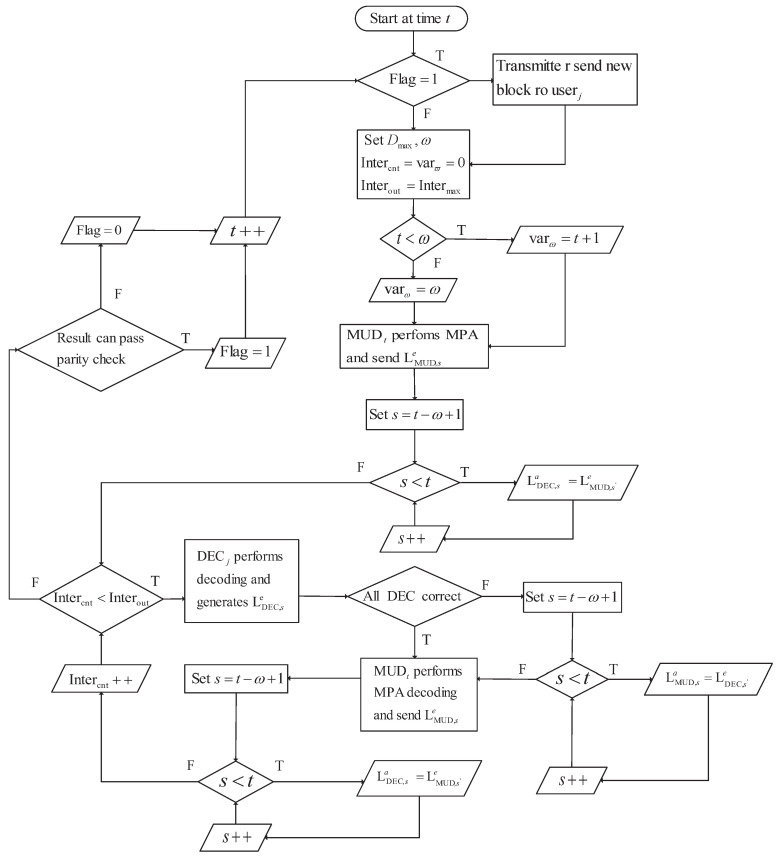
The procession of the *j*th user decoding at the time unit *t*.

The detailed windowed joint detection and decoding algorithm is given in Algorithm 1. Additionally, the functionality of this scheme for the user *j* at time unit *t* during operation is illustrated in Figure 4.

**Figure 5:**
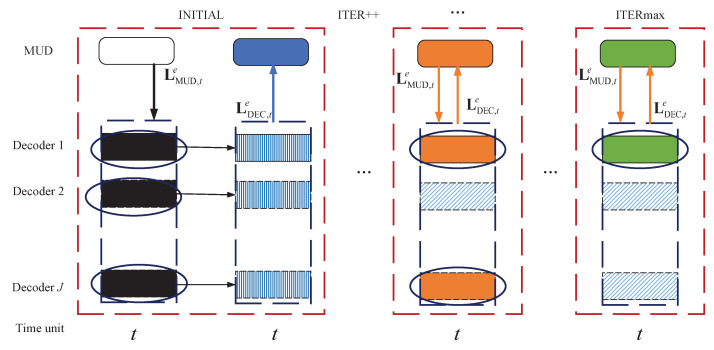
Decoding diagram with the original joint detection and decoding algorithm.

In addition, if the window size is set to w=1, then the iteration process between ${\mathrm{MUD}}_{s}$$\left({\mathrm{MUD}}_{t},\ldots ,{\mathrm{MUD}}_{t-w+1}\right)$ and ${\mathrm{DEC}}_{t}$ reduces to the original joint detection and decoding algorithm discussed in this paper. We can see the details in Figure 5, where the slashed rectangles indicate successfully decoded blocks, and the vertically lined rectangles represent blocks that failed to be decoded. The traditional joint detection and decoding algorithm, as shown in Figure 5, performs the iterations between the decoder and detector at the same time unit. However, the windowed joint detection and decoding algorithm exchanges the information between the detector and decoder not only at the current time units but also at the previous *w* time units, resulting in a noticeable improvement in complexity. Although achieving performance improvement comes at the cost of complexity, it is a worthy trade-off for the decoding algorithm for the system with HARQ .
**Algorithm 1** Windowed Joint Detection and decoding Algorithm.1:Initialization: $D_{\max}=4$, w=3, ${\mathrm{Iter}}_{\mathrm{out}}=5$, ${\mathrm{Iter}}_{\mathrm{cnt}}=0$, ${\mathrm{var}}_{w}=0$.2:**if** time unit t<w **then**3:   ${\mathrm{var}}_{w}=t+1$.4:**else**5:   ${\mathrm{var}}_{w}=w$.6:**end if**7:${\mathrm{MUD}}_{t}$ performs the MPA multi-user detection and sends the extrinsic information to ${\mathrm{DEC}}_{t}$ according to (7);8:**while** ${\mathrm{Iter}}_{\mathrm{cnt}}\;<\;{\mathrm{Iter}}_{\mathrm{out}}$ **do**9:   **for** j=1,2,…,J **do**10:     According to the current column and row indexes of the parity matrix, start the incremental decoding in ${\mathrm{DEC}}_{j}$:11:     **if** decoded correct **then**12:        Continue.13:     **else**14:        Save the extrinsic information;15:     **end if**16:   **end for**17:   **if** All the users decode correctly **then**18:     break;19:   **else**20:     ${\mathrm{DEC}}_{i}$ sends the extrinsic information to the detector ${\mathrm{MUD}}_{i}$, i=t,t−1,…,t−w+1 according to (6);21:   **end if**22:   ${\mathrm{MUD}}_{t},\ldots ,{\mathrm{MUD}}_{t-w+1}$ carry on the MPA multi-user detection separately;23:   MUDs deliver the extrinsic LLRs to the ${\mathrm{DEC}}_{t}$ according to (6).24:   ${\mathrm{Iter}}_{\mathrm{out}}++$;25:**end while**26:**for**j=1,2,…,J**do**27:   **if** decoded correct **then**28:     Send ACK to the transmitter ; hence, the new initial block will be sent.29:     Update current column and row indexes.30:   **else**31:     Update current column and row indexes.32:     Send NACK to the transmitter ; hence, the corresponding redundant parity bits will be sent.33:   **end if**34:**end for**

## 4. Simulation Results

To demonstrate the effectiveness of the proposed algorithm, we compare the performance of the original joint detection and decoding algorithm (According to Section 1.2, limited studies have been conducted on the decoding algorithm for the SCMA system with HARQ in recent years. Additionally, the innovative approaches presented in [18,19] are designed for the SCMA system with CC-HARQ, not the SCMA system with IR-HARQ), which can be obtained by setting the window size to w=1, with the performance of the proposed algorithm.

Considering the SCMA system with an RC-LDPC encoder, the information length is k=256, and the block length transmitted each time is I=400. Then, the initial block length and code rate are $N_{0}=400$ and $R_{0}=0.64$. We assume that there are J=6 users sharing L=4 resource elements. The maximum number of transmissions for one information block is $D_{\max}=4$, and the window size changes from w=1 to w=4.

**Figure 6:**
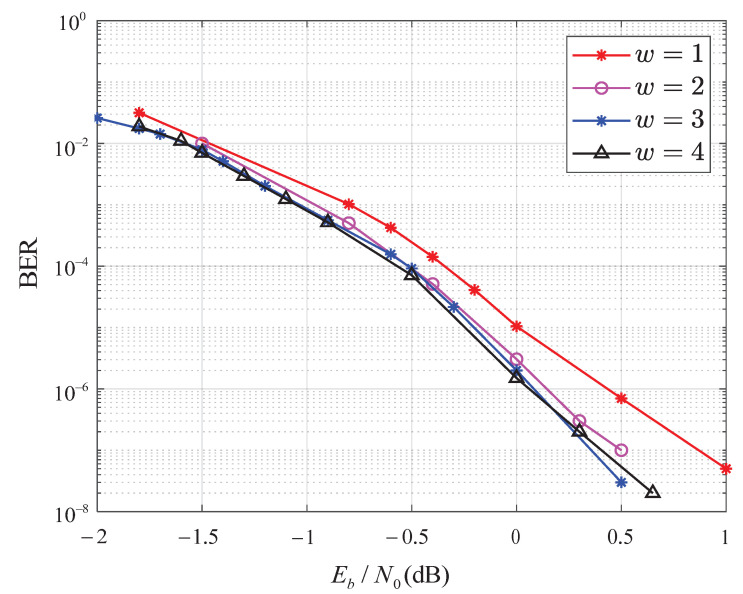
BER performance of the SCMA system with the IR-HARQ scheme using the windowed joint detection and decoding algorithm with $D_{\max}=4$.

Figure 6 shows the BER performance comparison of the SCMA system with two detection and decoding algorithms: traditional joint detection and decoding and windowed joint detection and decoding. The system uses a four-point, high-dimensional constellation over the additive white Gaussian noise (AWGN) channel, with an outer iteration of ${\mathrm{Iter}}_{\mathrm{out}}=3$ and an inner iteration of ${\mathrm{Iter}}_{\mathrm{in}}=5$. It is shown in Figure 6 that with the increase in *w*, the BER performance of windowed joint detection and decoding algorithm improves. In addition, when *w* is large enough (w=3 here), the BER performance improves slightly with the increase in *w*. We also see that the SCMA system using the windowed joint detection and decoding algorithm with w=3 outperforms the traditional joint detection and decoding algorithm, achieving about a 0.5 dB improvement in BER performance.

**Figure 7:**
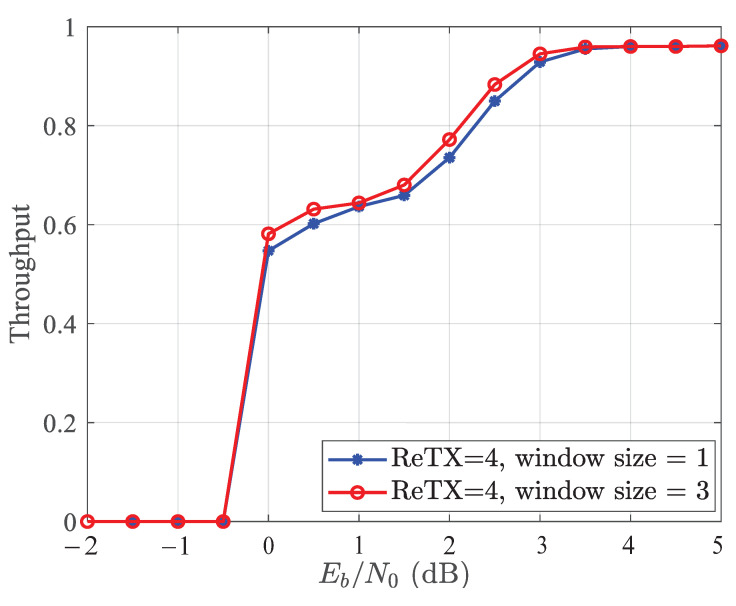
Throughput of the SCMA system with the IR-HARQ scheme using the windowed joint detection and decoding algorithm with $D_{\max}=4$.

Figure 7 compares the throughput of the traditional joint detection and decoding algorithm and the windowed joint detection and decoding algorithm. The throughput is denoted as the actual rate of RC-LDPC codes used to decode the fixed information blocks correctly. At low SNRs, both performances approach zero, while at high enough SNRs, both are good. The comparison shows that the SCMA system with the proposed algorithm has better throughput.

## 5. Conclusions

This paper introduces a new transmission strategy for the SCMA system using an RC-LDPC code and IR-HARQ. Additionally, we propose a windowed joint detection and decoding algorithm. The simulation results demonstrate that this scheme outperforms the original joint detection and decoding algorithm by approximately 0.5 dB and achieves a higher throughput across a wide range of SNRs.

## Data Availability

No new data were created or analyzed in this study. Data sharing is not applicable to this article.

## Abbreviations

The following abbreviations are used in this manuscript:

RC	rate-compatible
IR	incremental redundancy
SCMA	sparse code multiple access
HARQ	hybrid automatic repeat quest
AWGN	additive white Gaussian noise
NOMA	non-orthogonal multiple access
PD-NOMA	power-domain non-orthogonal multiple access
IDMA	interleave division multiple access
MUSA	multi-user shared access
PDMA	pattern division multiple access
LDS	low-density signature
QAM	quadrature amplitude modulation
MPA	message passing algorithm
UDCG	uniquely decomposable constellation group
BER	bit error ratio
UEP	unequal error protection
MED	minimum Euclidean distance
5G	fifth-generation mobile communication technology
CC	Chase combining
eMBB	enhanced mobile broadband
LLR	log-likelihood ratio
MUD	multi-user detection
MPA	message-passing algorithm
